# Diameter Dependence of Planar Defects in InP Nanowires

**DOI:** 10.1038/srep32910

**Published:** 2016-09-12

**Authors:** Fengyun Wang, Chao Wang, Yiqian Wang, Minghuan Zhang, Zhenlian Han, SenPo Yip, Lifan Shen, Ning Han, Edwin Y. B. Pun, Johnny C. Ho

**Affiliations:** 1 College of Physics and Cultivation Base for State Key Laboratory, Qingdao University, Qingdao 266071, China; 2 Department of Physics and Materials Science, City University of Hong Kong, 83 Tat Chee Avenue, Kowloon, Hong Kong; 3 State Key Laboratory of Millimeter Waves, City University of Hong Kong, Kowloon, Hong Kong; 4 Shenzhen Research Institute, City University of Hong Kong, Shenzhen, 518057, P.R. China; 5 Department of Electronic Engineering, City University of Hong Kong, 83 Tat Chee Avenue, Kowloon, Hong Kong; 6 State Key Laboratory of Multiphase Complex Systems, Institute of Process Engineering, Chinese Academy of Sciences, Beijing, 100190, P.R. China

## Abstract

In this work, extensive characterization and complementary theoretical analysis have been carried out on Au-catalyzed InP nanowires in order to understand the planar defect formation as a function of nanowire diameter. From the detailed transmission electron microscopic measurements, the density of stacking faults and twin defects are found to monotonically decrease as the nanowire diameter is decreased to 10 nm, and the chemical analysis clearly indicates the drastic impact of In catalytic supersaturation in Au nanoparticles on the minimized planar defect formation in miniaturized nanowires. Specifically, during the chemical vapor deposition of InP nanowires, a significant amount of planar defects is created when the catalyst seed sizes are increased with the lower degree of In supersaturation as dictated by the Gibbs-Thomson effect, and an insufficient In diffusion (or Au-rich enhancement) would lead to a reduced and non-uniform In precipitation at the NW growing interface. The results presented here provide an insight into the fabrication of “bottom-up” InP NWs with minimized defect concentration which are suitable for various device applications.

In recent years, due to the impressive electrical and optical properties, III–V semiconductor nanowires (NWs) are widely explored as promising active materials in the areas of electronics, photonics, life sciences and so on^1^,^2^,^3^,^4^,^5^,^6^,^7^. In particular, InP NWs have attracted extensive research interests with applications in future high-performance solar cells, photodetectors, field-effect transistors (FETs) and so on^4^,^8^,^9^,^10^ because of their direct and suitable bandgap for efficient photon coupling, excellent carrier mobility and wave-guiding characteristics^11^,^12^,^13^. Typically, high-quality InP NWs are prepared by metal-organic chemical vapor deposition (MOCVD), metal-organic vapor phase epitaxy (MOVPE) or molecular beam epitaxy (MBE) via catalytic vapor-liquid-solid (VLS) and/or vapor-solid-solid (VSS) mechanisms^10^,^14^,^15^,^16^. Despite recent progress in NW synthesis, the as-grown InP NWs usually still exhibit significant amount of planar defects and mixed crystal phases of hexagonal wurtzite (WZ) and cubic zincblende (ZB) structures along their growth directions, and are attributed to the low stacking fault energy and small formation energy difference between ZB and WZ structures in the nanoscale, respectively^17^,^18^,^19^. According to both theoretical calculations and experimental results, all these crystal defects and mixed phases would detrimentally influence the physical properties of InP NWs^19^,^20^,^21^. For instance, the intensity of room temperature photoluminescence of InP NWs is observed to be highly dependent on the extent of twin boundaries possessed^21^. When fabricated into transistors and photoelectrochemical cells, the electron mobility and open-circuit photovoltage are found to degrade severely with the defect concentration of InP NWs^19^,^21^. Hence, it is important to realize InP NWs with controllable defect density, which would further improve their excellent properties for various technological applications.

However, minimizing these crystal and phase defects in InP NWs has always been a challenging task. To date, much work has been implemented with the aim to fabricate high-quality InP NWs with tailorable morphology, perfect crystal structure as well as excellent electronic, optical and optoelectronic performances^13^,^17^,^22^,^23^,^24^,^25^. It has been shown that the morphology and crystal quality of InP NWs can be controlled by tuning the growth conditions, such as the growth temperature and/or the precursor V/III ratio^17^,^23^,^24^,^25^; but there are still few reports investigating the effect of NW diameter on the InP NW growth^26^,^27^,^28^, which is crucial for improving the NW crystallinity, carrier mobility and lifetimes. In our previous work, a facile and low-cost growth technique was developed to synthesize crystalline, dense and stoichiometric InP NWs on amorphous SiO_2_ substrates employing the VLS mechanism with Au nanoparticles as catalytic seeds^13^. Although the NWs grown contain a substantial amount of planar defects, the fabricated NW FETs exhibited excellent electrical performances with high I_ON_/I_OFF_ current ratio and high electron mobility. More importantly, these NWs can be contact-printed as regular parallel arrays and heterogeneously integrated on any substrates, demonstrating their promising potentials for practical deployments as compared to the more costly MBE or MOCVD grown counterparts with crystalline underlying substrates^5^,^29^,^30^. In this work, we investigate further the structural and chemical investigation of the Au-catalyzed InP NWs grown on amorphous substrates^31^,^32^,^33^,^34^. It is found that the planar defect density would decrease linearly with decreasing NW diameters, spanning from 50 to 10 nm, which can be attributed to the faster and more efficient In precursor diffusion in the smaller catalytic seeds during the VLS NW growth, due to the higher degree of In supersaturation associated with the Gibbs-Thomson effect. In larger NW diameters with lower In catalytic supersaturation, an insufficient In diffusion (or Au-rich enhancement) would lead to a reduced and non-uniform In precipitation at the NW growing interface, yielding a significant quantity of planar defects. Our findings provide a valuable insight into the fabrication of “bottom-up” InP NWs with minimized defect concentration, confirming the careful consideration of the NW diameter and structure is required, and show the way for achieving enhanced crystallinity and subsequent optimized device performances.

## Results

**Figure 1:**
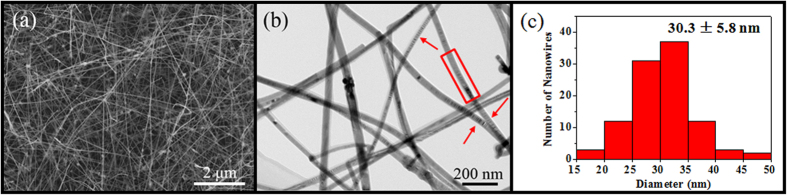
(**a**) SEM image of high-density InP NWs grown on amorphous Si/SiO_2_ substrates. (**b**) Bright field TEM image of InP NWs with planar defects indicated by the red box and arrows. (**c**) Diameter statistics of more than 100 individual NWs observed in the corresponding bright field TEM images.

As shown in the SEM image (Fig. 1a), the synthesized InP NWs are relatively dense, straight and long with typical lengths greater than 10 μm. Further investigations using TEM reveal that all NWs are grown with smooth surfaces and uniform diameters along their growth directions (Fig. 1b). Based on the statistics of more than 100 individual NWs, the NW diameters are found to range from 15 to 50 nm with an average value of 30.3 ± 5.8 nm when the thickness of the amorphous native oxide layer (3–5 nm) is concluded (Fig. 1c, discussed in the following section). This relatively wide distribution of diameters obtained from single growth run ensures a consistent study of the effect of NW diameters on the formation of crystal defects. It is obvious that there are several alternating bright/dark contrast fringes appearing in a periodic manner along the NW axial direction as designated by the red arrows, which indicate the existence of planar defect structures studied in this work^20^,^35^.

**Figure 2:**
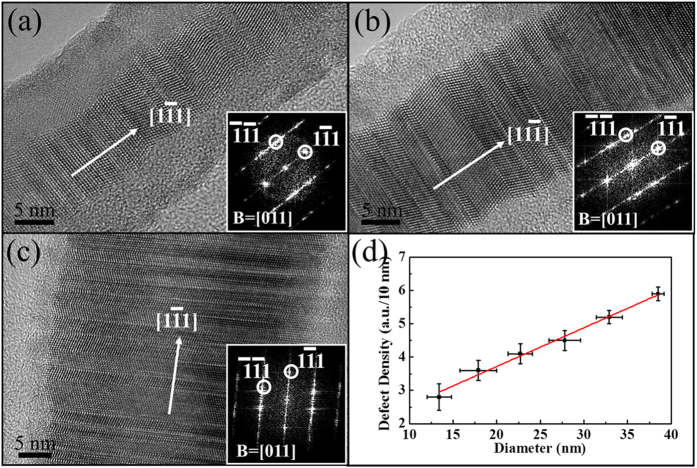
(**a**–**c**) High-resolution TEM images of InP NWs in their body regions with diameters of ~11 nm, 20 nm and 39 nm, respectively, and the thickness of the amorphous native oxide layer has been subtracted from the NW diameter determination. Insets in (**a**–**c**) are the corresponding FFT images of the NWs, which indicate the ZB structures of NWs here. (**d**) The statistical compilation of the planar defect density per 10 nm along the NW growth direction as a function of the NW diameter. All the results are based on the information extracted from the TEM images.

High-resolution TEM is used to further systematically evaluate the relationships between crystal structures, chemical composition and planar defect characteristics of the obtained InP NWs, and the corresponding images of three representative NWs with different diameters of ~11, 20 and 39 nm are shown accordingly (Fig. 2a–c). It should be noted that there is always an amorphous native oxide layer of InO_x_ with a thickness of 3–5 nm surrounding the NWs (see Supplementary Fig. S1), which is a general phenomenon for VLS grown III–V NWs because the Gibbs formation energy of oxides are far below that of III–V compounds and thus residual oxygen would oxidize the NW surface once exposing to the ambient^36^. However, as compared with other III–V NWs such as InAs, GaAs, GaP and GaSb synthesized by the similar chemical vapor deposition method^5^,^6^,^7^,^37^,^38^, there are insignificant amounts of planar defects existed in the NWs, even though a similar thickness of native oxide layers is also observed there. In this case, we believe that the native oxide layer has a negligible effect on the formation of planar defects in InP NWs, which is different from the effect of oxygen in other metal-oxide semiconductor NWs, where the deficiency of oxygen would lead to the formation of oxygen vacancies as point defect and planar defects would as well be formed if the delocalized oxygen atoms were removed along the periodic distance^39^,^40^. This layer thickness is deducted from all the diameter values discussed in this investigation. Combining with the FFT images (inset of Fig. 2a–c), all these NWs exhibit ZB crystal structure and preferential growth orientation along the <111> direction, in which these pure crystal phase and uniform growth direction are necessary for large-scale device fabrication. As shown in the images, the NWs also contain significant amount of randomly distributed stacking faults and twin boundaries along the NW growth direction, which are consistent with the bright/dark contrast fringes as shown in Fig. 1b along the NW axial direction. Majority of the planar defects of InP NWs studied here are characterized as twin planes (>65%), which can be attributed to the relatively low formation energy of twin planes, approximately equal to the half the stacking fault energy, that are ~18 mJ/m^2^ and ~9 mJ/m^2^, respectively^18^,^41^,^42^. Since the planar defect energies of InP NWs are comparably low among all typical III–V NWs (e.g. InAs, GaP and others), planar defects would be easily formed during the NW growth, in which these planar defects are found to be reduced by carefully tuning the experimental parameters such as the NW diameter and In supersaturation of the Au_x_In_y_ catalysts. More importantly, these planar defect densities increase with increasing NW diameters. To quantify this observation, we define the planar defect density as the average number of planar defects per 10 nm length of NW. Based on the compilation of more than 30 NWs and a minimum examination length of 500 nm for each NW, the planar defect density is found to increase linearly as a function of InP NW diameter with d = 10–40 nm with a slope of ~0.12 (a.u./10 nm)/nm (Fig. 2d). Larger or smaller diameters beyond the range reported here were not investigated due to the difficulty employing our growth conditions^13^.

The linear increase in the planar defect density with increasing NW diameter can be attributed to a number of factors. It has been demonstrated by both theoretical calculations and experiments that the NW crystal structure, whether existing in the WZ or ZB phases, is not only depended on the chemical composition and growth conditions^14^,^25^, but also dictated by the NW diameter, in which thin NWs prefer the formation of WZ phase, while thick NWs tend to grow with ZB structure^14^,^42^. Notably, since the InP NWs are grown non-epitaxially on amorphous substrates here, there is not any underlying crystalline substrate guiding the initial NW growth. However, as InP {111} planes are the close-packed surface with the lowest free energy in the ZB structure, the NW growth on {111} planes along the <111> directions are favorable energetically; therefore, the NW nuclei align with this orientation would always grow faster and tend to dominate during the growth^14^,^43^. In addition, as confirmed by the TEM observations, InP NWs within the above-mentioned diameter range are always grown with the ZB structure in the bulk phase, but they often contain rotational blocks which are common in the growth of ZB III–V NWs, which is caused by the small energy difference between the normal and twin nuclei^14^,^44^. Since their rotational axes also align well along the <111> directions, these blocks would then result in planar defects such as stacking faults and twin boundaries, accordingly. Thus, all these evidences illustrate the formation of planar defects in the InP NWs, and works have been carried out to further understand these defects.

**Figure 3:**
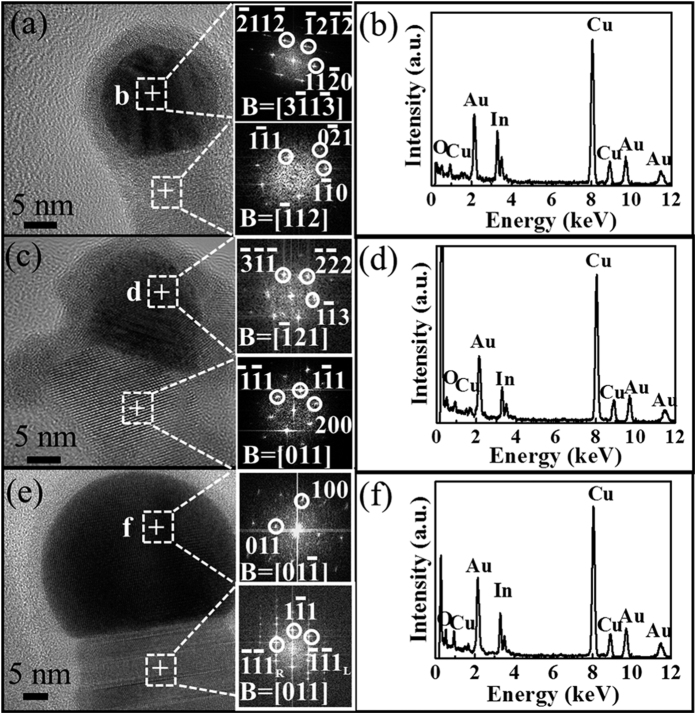
(**a,c,e**) High-resolution TEM images of representative InP NWs with different diameters of ~11, 18 and 39 nm, resepectively. The top and bottom insets give the FFT images of the catalyst tip and NW body, accordingly. (**b,d,f**) EDS spectra of the catalyst tip as shown in the panel of (**a**,**c**,**e)**, respectively.

In an effort to examine the origin of the diameter dependent planar defects in synthesized InP NWs, extensive investigations were carried out. As shown in the BF TEM images (see Supplementary Fig. S1a–c) and the corresponding high-resolution TEM images of the NW tip region (Fig. 3a,c,e), Au-based spherical catalytic tips are clearly observed at one ends of the NWs, which suggests the presence of VLS growth mechanism in the NWs and are consistent with the previous reports^10^,^12^,^13^,^14^. Moreover, according to the VLS growth mode, since the NW growth is initiated at the catalyst/NW interface, the catalytic supersaturation is expected to have an overwhelming influence over the growth^37^,^43^,^45^. Therefore, it is important to assess the chemical composition and the crystal structure of the catalytic tips with different NW diameters. The catalysts are mainly composed of Au and In with estimated atomic ratios of 3:2, 9:4 and 3:1 for the thinnest, medium and thickest group of NWs (i.e. average d~11, 18 and 39 nm), respectively, and no P is detected in the EDS measurements (Fig. 3b,d,f). The lattice spacing determined from the TEM and corresponding FFT images (inset of Fig. 3a,c,e) indicate that the catalysts exist in the hexagonal Au_3_In_2_ phase (PDF 00-026-0710) with lattice parameters of a = 0.456 nm, c = 0.907 nm, cubic Au_9_In_4_ phase (PDF 00-029-0649) with lattice parameters of a = 0.984 nm and orthorhombic Au_3_In phase (PDF 03-065-2592) with lattice parameters of a = 0.586 nm, b = 0.475 nm and c = 0.517 nm, accordingly, and all these are consistent with the EDS results. It is noted that the In content of the catalytic tips is found to increase with the decreasing NW diameter, suggesting that smaller Au_x_In_y_ catalysts can yield the higher In supersaturated catalyst tips which can produce thinner InP NWs with the fewer planar defects, while larger Au_x_In_y_ catalysts with the lower In supersaturation can only induce thicker InP NWs with the higher density of planar defects. In addition, the amount of planar defects is found independent on the crystal structure of Au_x_In_y_ catalysts, because the InP NWs were typically grown by VLS mechanism. In specific, during the NW growth, catalysts existing in the liquid state do not have any crystal structure to epitaxially dictate the crystal phase and growth orientation of NWs^13^. As a result, the amount of planar defects is monotonically increased with increasing the NW diameters (Fig. 2d), while it increases with decreasing In content of the catalytic tip (see Supplementary Fig. S2).

**Figure 4:**
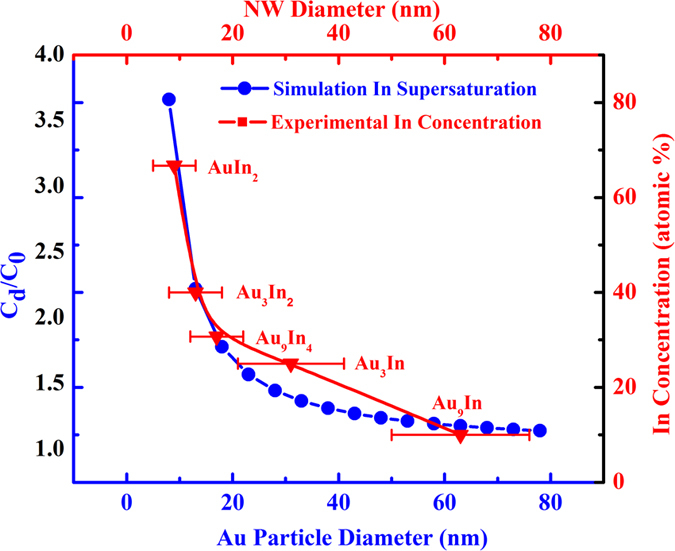
Simulation of the catalytic supersaturation of In in Au nanoparticles with different diameters (blue line), and the experimental results of catalytic In concentration with different NW diameters (red line).

Further theoretical analysis of the catalytic supersaturation discussed above is needed in order to understand the role of In supersaturation in the Au nanoparticles, and their possible relationships to the planar defect formation during the NW growth. Based on the Gibbs-Thomson effect, it is well established that Au particles can be supersaturated by In in the nanometer scale due to the larger surface energy of nanoparticles, which can be described by the equation (1)
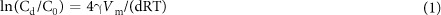
where C_d_ is the concentration of In in Au nanoparticle with diameter of *d*, C_0_ is the equilibrium concentration in flat surface (d → ∞) materials, γ is surface energy (1.14 J m^−2^), V_m_ is molar volume of Au (assuming in molten phase, 1.14 × 10^−5^ m^3^ mol^−1^), R is constant (8.314 J mol^−1^ K^−1^), and T is the growth temperature (constant at 733 K for all our growth trials)^46^. By varying the Au particle diameter, this equation would generate an In supersaturation curve (blue colored line with solid circular solid dots in Fig. 4). Comparing our experimental results of 66.7 (AuIn_2_), 40 (Au_3_In_2_), 30.7 (Au_9_In_4_), 25 (Au_3_In) and 10 (Au_9_In) atomic % In concentration in different NW diameters (red colored line with solid triangular dots) to the simulated curve, the experimental results agree well with the simulation results for the exponential increase of In supersaturation in decreasing diameter NWs, assuming a C_0_ of 10 atomic % In concentration. The slight discrepancy between the experimental and simulated results observed here can be ascribed to the difference between the simulated spherical Au diameters versus the actual hemispherical sizes.

## Discussion

**Figure 5:**
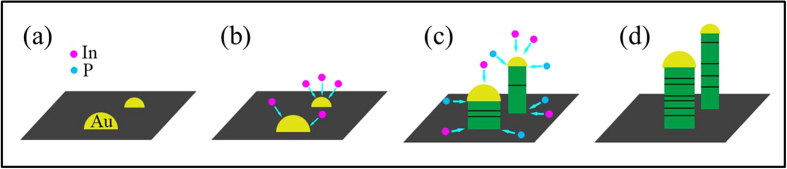
Schematic illustration of the VLS growth mechanism of Au-catalyzed InP NWs in our studies. (**a**) Formation of Au nanoparticles from the 0.5 nm thick Au catalyst film after annealing; (**b**) Au_x_In_y_ alloy seeds are formed by the diffusion of In atoms into the Au catalyst nanoparticles; the In concentration becomes higher for the smaller particles while the In concentration is lower for the larger particles; (**c**) InP NWs are grown by supplying P constituents to the catalyst/NW interface and then reacting with In from supersaturation in the catalytic tips; the planar defect density is lower for narrow NWs and becomes higher for thick NWs; (**d**) continuous axial growth of the InP NWs with different NW diameters and defect densities.

Based on the detailed characterization and analysis outlined, smaller catalysts are found to yield higher supersaturated catalyst tips and also thinner InP NWs with fewer planar defects. The diameter dependent planar defect formation could be explained as follows (Fig. 5). When the Au catalyst film (i.e. 0.5 nm in the nominal thickness) is annealed, a wide distribution of nanoparticle sizes can be obtained (Fig. 5a). With decreasing catalyst size, the corresponding surface-to-volume ratio would increase rapidly, making the In precursor elements to diffuse much faster into the catalyst particles (Fig. 5b)^43^,^45^,^47^. The InP NWs are grown by supplying P constituents to the catalyst/NW interface (Fig. 5c), rather than dissolving in the catalyst or alloying with the Au_x_In_y_ nanoparticles due to its relatively low solubility in Au^48^, which also explains the absence of P feature peak in the EDS spectrum in the catalytic tip (Fig. 3). Moreover, the high In content in the catalyst would induce higher catalytic supersaturation, contributing to the continuous, efficient and homogenous precipitation of In from the liquid Au alloy tips to chemically react with the P precursor. Thus, the reacted products would be able to effectively migrate themselves into the appropriate atomic sites to yield InP NWs with lower planar defect density. On the other hand, for larger NW diameters with lower In catalytic supersaturation, an insufficient In diffusion (i.e. Au-rich enhancement) would lead to reduced and non-uniform In precipitation at the NW growing interface, resulting in a significant amount of planar defects there (Fig. 5d). Similar phenomena have also been observed in Au-catalyzed GaAs and InAs NWs^43^,^45^. The EDS spectra reveal that the atomic ratio of In:P is approximately 1:1 in the NW body region for all the diameters studied (see Supplementary Fig. S1d,e,f), indicating that the obtained InP NWs are stoichiometric despite the existence of planar defects. All these results provide a valuable insight into the realization of low-defect InP NWs for practical applications.

In summary, InP NWs with pure cubic zincblende phase and uniform growth orientation have been successfully synthesized via a facile and low-cost solid-source catalytic chemical vapor deposition on amorphous substrates. Based on the detailed structural characterization, a significant amount of planar defects, such as stacking faults and twin boundaries, are clearly observed along the NW growth direction of <111>. Specifically, these defects are found to linearly increase with NW diameter for d = 10–40 nm. This dependence can be explained by the higher degree of In supersaturation in the smaller Au catalyst nanoparticles due to the Gibbs-Thomson effect, and the In precursor can be homogenously incorporated within the particles and react with the P constituent more effectively to yield NWs with minimized planar defects. All these findings enable further understanding in the synthesis of high-quality InP NWs, and with careful NW diameter and structural design considerations, enhanced crystallinity and optimized device performances can be obtained.

## Method

### Synthesis of InP NWs

InP NWs used in this study were prepared by a solid-source catalytic chemical vapor deposition method (SSCVD) in a dual-zone horizontal tube furnace as previously reported^13^. In brief, the solid source (1 gram, InP powder, 99.995% purity) was placed in a boron nitride crucible and evaporated at the center of the upstream zone, while the substrate (Si/SiO_2_ pieces with the 50 nm thick thermally grown oxide) was placed in the middle of the downstream zone with a tilt angle of approximately 20° and a distance of 10 cm away from the source. Au catalyst films with a nominal thickness of 0.5 nm were pre-deposited in a thermal evaporator under a vacuum of approximately 1 × 10^−6^ Torr onto the substrates followed by thermally annealing at 800 °C for 10 min in a hydrogen environment (99.999% purity) in order to obtain Au nanoclusters in the downstream zone. This method provides a simple and cost-efficient approach in the preparation of Au catalysts which are further catalyzed to grow high quality NWs. When the substrate temperature was cooled to the preset growth temperature of 460 °C, the source was heated to the required source temperature of 770 °C. H_2_ with a flow rate of 100 sccm was used as the carrier gas to transport the precursor vapor to the substrate for the entire growth duration of the 30 min under a process pressure of 1 Torr. After growth, the source and substrate heaters were switched off, and the substrates are cooled down to room temperature under H_2_ ambient.

### Characterization of InP NWs

Morphologies of the grown InP NWs were examined by scanning electron microscope (SEM, Philips XL30) and bright-field (BF) transmission electron microscopy (TEM, Philips CM-20). Crystal structures were determined by studying the images and the reciprocal lattice spots extracted from the Fast Fourier Transform (FFT) with a high-resolution TEM (JEOL 2100F, operating at 200 kV). The chemical composition of the grown NWs and the catalyst tips were analyzed by energy dispersive X-ray (EDX) detector attached to JEOL 2100F. Specimens for TEM examinations were prepared by peeling off the InP NWs from the substrate surfaces, suspending them in anhydrous ethanol by ultrasonication for several seconds, and then dispersing the final solution onto holey-carbon-film-coated copper grids.

## Additional Information

**How to cite this article**: Wang, F. *et al*. Diameter Dependence of Planar Defects in InP Nanowires. *Sci. Rep.* **6**, 32910; doi: 10.1038/srep32910 (2016).

## Supplementary Material

Supplementary Information
