# Supplementary material for: Diameter Dependence of Planar Defects in InP Nanowires
Source: Sci Rep. 2016 Sep 12;6:32910. doi: 10.1038/srep32910 (PMC5018732; doi:10.1038/srep32910)
Supplement: Supplementary Information [file srep32910-s1.pdf]

Diameter dependence of planar defects in InP nanowires

Fengyun Wang[1,2*], Chao Wang[1], Yiqian Wang[1], Minghuan Zhang[1], Zhenlian Han[1], SenPo Yip[2,3,4], Lifan Shen[3,4,5], Ning Han[6], Edwin Y.B. Pun[3,5], Johnny C. Ho[2,3,4,*]

[1]College of Physics and Cultivation Base for State Key Laboratory, Qingdao University, Qingdao 266071, China

[2]Department of Physics and Materials Science, City University of Hong Kong, 83 Tat Chee Avenue, Kowloon, Hong Kong

[3]State Key Laboratory of Millimeter Waves, City University of Hong Kong, Kowloon, Hong Kong

[4]Shenzhen Research Institute, City University of Hong Kong, Shenzhen, 518057, P.R. China

[5]Department of Electronic Engineering, City University of Hong Kong, 83 Tat Chee Avenue, Kowloon, Hong Kong

[6]State Key Laboratory of Multiphase Complex Systems, Institute of Process Engineering, Chinese Academy of Sciences, Beijing, 100190, P.R. China

* Author to whom correspondence should be addressed.

Electronic mail: johnnyho@cityu.edu.hk; fywang@qdu.edu.cn

SUPPLEMENTARY INFORMATION

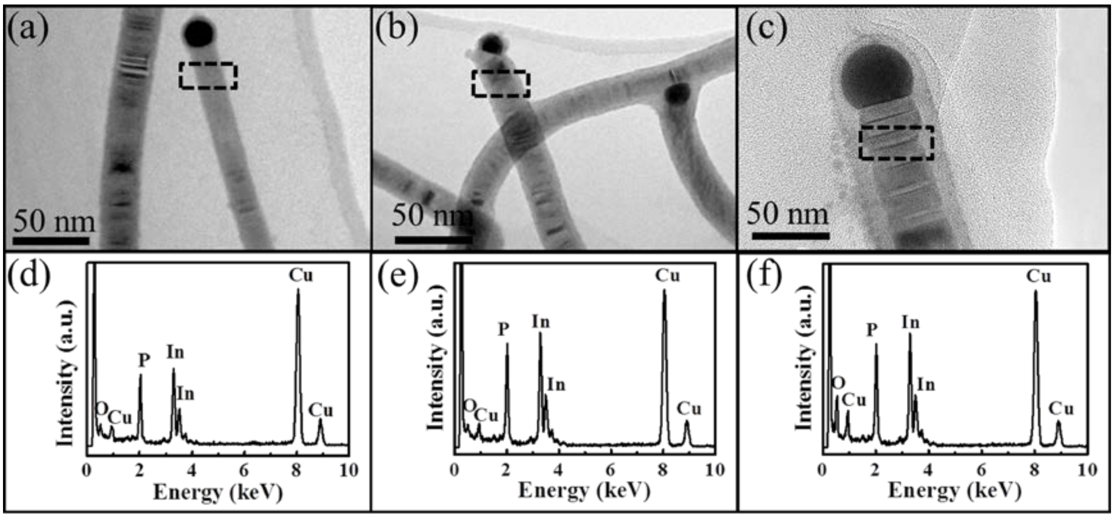

Figure S1. Bright field TEM images of typical InP NWs with different diameters of ~ 11 nm (a), ~ 18 nm (b) and ~ 39 nm (c); (d), (e) and (f) are the EDS spectra of the NW body shown in the panel of a, b and c, respectively.

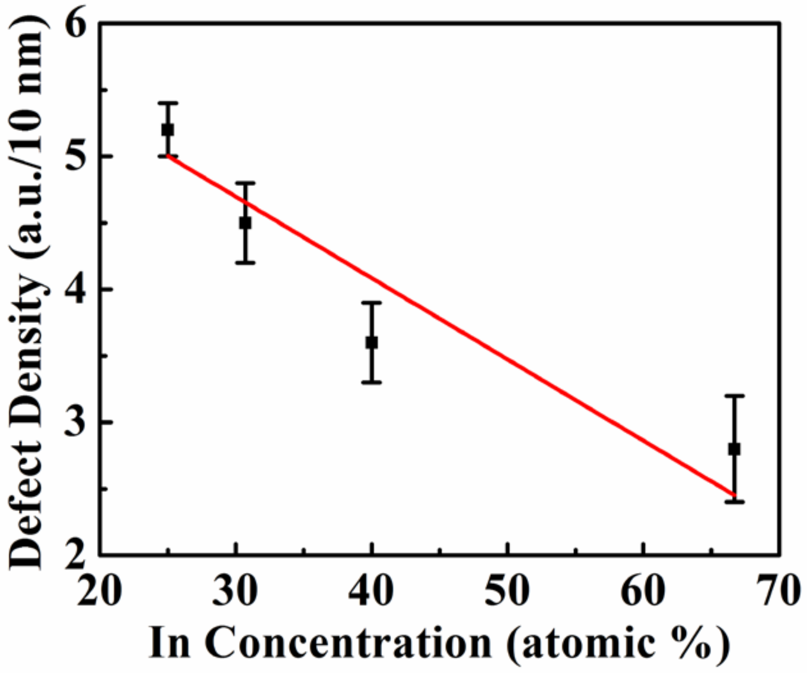

Figure S2. Statistical results of the planar defect density per 10 nm along the NW growth direction as a function of the In content of the Au_xIn_y catalysts. All these results are extracted based on the HRTEM images obtained in this work.
